# Facilely reducing recalcitrance of lignocellulosic biomass by a newly developed ethylamine-based deep eutectic solvent for biobutanol fermentation

**DOI:** 10.1186/s13068-020-01806-9

**Published:** 2020-10-09

**Authors:** Guochao Xu, Hao Li, Wanru Xing, Lei Gong, Jinjun Dong, Ye Ni

**Affiliations:** 1 Key Laboratory of Industrial Biotechnology, Ministry of Education, School of Biotechnology, Jiangnan University, Wuxi, 214122 Jiangsu China; 2 Key Laboratory of Guangxi Biorefinery, Nanning, 530003 Guangxi China

**Keywords:** Deep eutectic solvent, Lignocellulosic biomass, Ethylamine, Pretreatment, Biobutanol

## Abstract

**Background:**

Biobutanol is promising and renewable alternative to traditional fossil fuels and could be produced by *Clostridium* species from lignocellulosic biomass. However, biomass is recalcitrant to be hydrolyzed into fermentable sugars attributed to the densely packed structure by layers of lignin. Development of pretreatment reagents and processes for increasing surface area, removing hemicellulose and lignin, and enhancing the relative content of cellulose is currently an area of great interest. Deep eutectic solvents (DESs), a new class of green solvents, are effective in the pretreatment of lignocellulosic biomass. However, it remains challenging to achieve high titers of total sugars and usually requires combinatorial pretreatment with other reagents. In this study, we aim to develop novel DESs with high application potential in biomass pretreatment and high biocompatibility for biobutanol fermentation.

**Results:**

Several DESs with betaine chloride and ethylamine chloride (EaCl) as hydrogen bond acceptors were synthesized. Among them, EaCl:LAC with lactic acid as hydrogen bond donor displayed the best performance in the pretreatment of corncob. Only by single pretreatment with EaCl:LAC, total sugars as high as 53.5 g L^−1^ could be reached. Consecutive batches for pretreatment of corncob were performed using gradiently decreased cellulase by 5 FPU g^−1^. At the end of the sixth batch, the concentration and specific yield of total sugars were 58.8 g L^−1^ and 706 g kg^−1^ pretreated corncob, saving a total of 50% cellulase. Utilizing hydrolysate as carbon source, butanol titer of 10.4 g L^−1^ was achieved with butanol yield of 137 g kg^−1^ pretreated corncob by *Clostridium saccharobutylicum* DSM13864.

**Conclusions:**

Ethylamine and lactic acid-based deep eutectic solvent is promising in pretreatment of corncob with high total sugar concentrations and compatible for biobutanol fermentation. This study provides an efficient pretreatment reagent for facilely reducing recalcitrance of lignocellulosic materials and a promising process for biobutanol fermentation from renewable biomass.

## Background

Biofuels are promising, renewable and natural alternatives to traditional fossil fuels and have gained great interest [1]. Among them, biobutanol possesses great potential due to its high hydrophobicity, high energy density, low corrosiveness and more compatibility in mixing with gasoline [2, 3]. Moreover, biobutanol could be produced by *Clostridium* species from lignocellulosic biomass including agricultural waters, forestry residues, grasses and woody materials, which are abundant and renewable resources on the earth. Generally, there are about 10‒25% lignin, 20‒30% hemicellulose, and 40‒50% cellulose in most agricultural lignocellulosic biomass, which can be converted into fermentable sugars, value-added fine chemicals and materials, etc. [4]. Nevertheless, most of agricultural lignocellulosic resources have been improperly disposed by open field burning. This common practice has led to the emission of pollutants such as CO_2_, CO, NO_X_, SO_2_, dioxins, etc. [5–7], resulting in air pollution and threatening public health [8, 9]. According to statistics, more than 800 million tons of agricultural lignocellulosic biomass has been produced annually in China since 2008, and only about half of the biomass was utilized as fertilizer or feed. Among them, corncob is one of the most important lignocellulosic materials with relatively higher contents of cellulose and hemicellulose and lower lignin amount, especially suitable for biofuels production [10, 11].

Although lignocellulosic biomass displays great potential in producing renewable energy, it is recalcitrant to be hydrolyzed into fermentable sugars attributed to the densely packed structure by layers of lignin [12, 13]. Pretreatment of biomass, aiming at increasing surface area, removing hemicellulose and lignin, and enhancing the relative content of cellulose, is required to enhance its accessibility to cellulases for conversion into fermentable sugars. An ideal pretreatment method should be efficient in removal of lignin and hemicellulose, cost-effective and energy-efficient, and highly biocompatible [4, 14]. There are various pretreatment methods, including alkali, acids, ionic liquids, organic solvents, thermal, pressure, etc. [15, 16]. Among them, acidic pretreatment methods have been intensively studied, possessing significant advantages of low-cost and high efficiency in destruction of biomass recalcitrance [17]. However, acids are corrosive to equipment and toxic to cellulases and microorganisms. Ionic liquids (ILs), generally consisted of hydrogen bond donor (HBD) and hydrogen bond acceptor (HBA), are promising reagents and have gained tremendous attention due to their low melting temperature, tunable combinations of various cation and anion, easy preparation, low vapor pressure, recyclability and biocompatibility [18]. Various ILs have been developed and applied in biomass pretreatment, such as [Bmim][Cl], [Bmin][H_2_SO_4_] or [Emim][Cl] [19–21]. However, traditional ILs are expensive and less effective in reducing the recalcitrance, usually requiring other combinatorial methods such as alkaline. Deep eutectic solvents (DESs) are also made up of HBD and HBA, and emerging as a new class of ILs with similar physical and chemical properties. Most importantly, DESs are advantageous due to their easy preparation, high stability, good biodegradability and biocompatibility [22–24]. Choline chloride (ChCl), a bulk chemical, based deep eutectic solvents was firstly synthesized by Abbott at 2004 [25]. Since then, various ChCl-based DESs have been developed and attempted in the pretreatment of various lignocellulosic biomass such as corn stover, corncob, rice straw, bamboo shell, etc., with high efficiency in removing lignin [3, 26–29]. Endeavor has been committed in optimizing the HBDs including organic or inorganic acids, amino acids, alcohols and sugars. In fact, ChCl-based DESs display similar or ever higher efficiency in reducing the recalcitrance of lignocellulosic biomass than traditional ILs such as [BMIM][Cl], attributing to the similar mechanism of providing or receiving H-bond or localizing charges on the chemical bonds of biomass through electrostatic forces of the ionic pairs [30]. However, high titers (> 50 g L^−1^) of fermentable sugars are hard to be achieved by single pretreatment with ChCl-based DESs. Generally, condensation under vacuum or combinatorial pretreatment methods such as NaOH, Na_2_CO_3_ or microwave radiation should be introduced, inevitably complicating the process and resulting in low solid yield and high consumption of water for removing residual alkaline. As a result, the utilization of lignocellulosic biomass with high yields and titers of total sugars remains challenging [31]. Development of novel DESs with enhanced properties in pretreating biomass is of significant interest for biofuels production from abundant and renewable biomass.

**Scheme 1.**
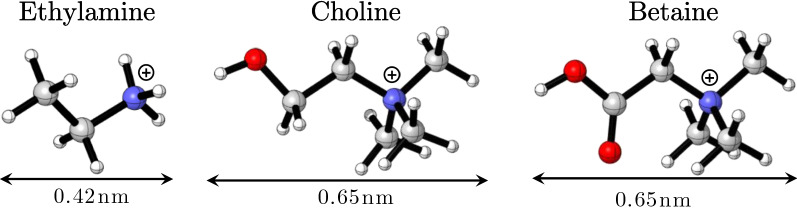
Structures of ethylamine chloride, choline chloride and betaine chloride used as hydrogen bond acceptors. Atoms are carbon (gray), nitrogen (blue), oxygen (red), and hydrogen (white). Cl^−^ ion is not shown

In principle, ammonium, phosphonium, or sulfonium cation in halide form could be used as HBAs, while amides, carboxylic and alcohols could be used as HBDs [32]. Development of novel DESs can be achieved by rational complexation of HBDs and HBAs to form eutectic liquids. However, the procedures are empirical and labor-intensive, and most combinations are unsuccessful in forming homogeneous and clear liquids. Density functional theory (DFT) calculations play an important role in elucidating the mechanism of chemical or enzymatic reactions and also predicting the reactivity [33, 34]. Here, DFT calculation was adopted to evaluate the feasibility in synthesis of novel DESs. Cheap and bulk chemicals including ChCl, betaine chloride (BaCl) and ethylamine chloride (EaCl) were investigated as HBAs (Scheme 1) for developing efficient DESs. Newly synthesized DESs were evaluated for removing of lignin and hemicellulose, as well as their effects on increasing cellulose accessibility of biomass. Corncob hydrolysates were also examined in biobutanol fermentation by *Clostridium saccharobutylicum* DSM13864.

## Results and discussion

### Synthesis of DESs

Betaine chloride (BaCl) and ethylamine chloride (EaCl) possess similar structure as ChCl, they were explored as HBA in the synthesis of DESs. As shown in Scheme 1, EaCl is smaller than ChCl, while BaCl is similar to ChCl. All of them contain quaternary nitrogen cation, which is favorable to DES formation. There is a free hydroxy group in ChCl, a carboxyl group in BaCl and a free aliphatic terminal in EaCl. Lactic acid (LAC), ethyl glycol (EG), glycerol (GLY) and urea (UR), which have been commonly used and proved to be effective in forming DESs with ChCl, were introduced as HBDs. Generally, DESs were empirically synthesized by combination of various kinds and ratios of HBD and HBA. However, most of the combinations were hard to be synthesized. Considering these unpredictable combination patterns, both experimental synthesis employing methods for ChCl-based DESs and DFT calculations with different functions were performed to explore the feasibility of rational design of DESs.

For ChCl and EaCl-based DESs, all of the four combinations, ChCl:LAC, ChCl:EG, ChCl:GLY and ChCl:UR, EaCl:LAC, EaCl:EG, EaCl:GLY and EaCl:UR, respectively, were successfully obtained. However, with regard to BaCl as HBA, only BaCl:EG and BaCl:GLY could be facilely synthesized in clear and homogenous liquid. It should be mentioned that further optimization of reaction conditions such as ratios of HBD to HBA and temperature might also produce BaCl:LAC and BaCl:UR. Furthermore, Δ*G*_rxn_ of the reaction (Δ*G*_rxn_ = *G*_DES_ − *G*_HBA_ − *n* × *G*_HBD_) was calculated employing three mostly common used DFT including B3LYP, M062X and ωB97X and basis set of 6 − 311 + G**. Negative values of Δ*G*_rxn_ can be used to indicate the thermodynamical feasibility. Previously, B3LYP and M062X have been used in the simulation of ChCl-based DESs [35–38]. In Table 1, Δ*G*_rxn_ values of easily obtained DESs were lower than 0, according to the Δ*G*_rxn_ results of B3LYP ($\Delta G_{{{\text{rxn}}}}^{{{\text{B3LYP}}}}$). Especially, the $\Delta G_{{{\text{rxn}}}}^{{{\text{B3LYP}}}}$ values of ChCl:LAC and EaCl:LAC were the lowest. With regard to BaCl:LAC and BaCl:UR, the $\Delta G_{{{\text{rxn}}}}^{{{\text{B3LYP}}}}$ values were 3.59 and 1.03 kcal mol^−1^, respectively. For the results using M062X, there is no definite patterns. For example, the $\Delta G_{{{\text{rxn}}}}^{{{\text{M}}0{\text{62X}}}}$ value of BaCl:UR was − 10.8 kcal mol^−1^, ranking the lowest, whereas, $\Delta G_{{{\text{rxn}}}}^{{{\text{B3LYP}}}}$ of BaCl:UR was the highest. All the $\Delta G_{{{\text{rxn}}}}^{{\upomega {\text{B}}97{\text{XD}}}}$ values were negative, which were hard to be correlated with the reactivity. As a result, calculation method of B3LYP/6 − 311 + G** is more favorable in predicting the potential of DES synthesis, and might be used to elucidate the reactivity and mechanism of DES mediated systems in pretreatment of lignocellulosic biomass.

**Table 1:** Deep eutectic solvents synthesized in this study

DES	Ratio of HBA to HBD	$\Delta G_{{{\text{rxn}}}}^{{{\text{B3LYP}}}}$ (kcal mol^−1^)	$\Delta G_{{{\text{rxn}}}}^{{{\text{M}}0{\text{62X}}}}$ (kcal mol^−1^)	$\Delta G_{{{\text{rxn}}}}^{{\upomega {\text{B}}97{\text{XD}}}}$ (kcal mol^−1^)
ChCl:UR	1:2	− 2.54	− 6.39	− 6.50
ChCl:EG	1:2	− 2.84	− 7.15	− 6.55
ChCl:GLY	1:2	− 2.27	− 2.86	− 2.58
ChCl:LAC	1:1	− 8.96	− 10.3	− 9.95
BaCl:UR	1:2	3.59	− 10.8	− 0.54
BaCl:EG	1:2	− 1.42	− 11.2	− 7.15
BaCl:GLY	1:2	− 1.21	− 9.88	− 1.73
BaCl:LAC	1:1	1.03	− 4.92	− 3.23
EaCl:UR	1:2	− 2.25	− 4.55	− 3.80
EaCl:EG	1:2	− 1.62	− 4.21	− 1.73
EaCl:GLY	1:2	− 2.38	− 4.19	− 3.31
EaCl:LAC	1:1	− 4.67	− 7.94	− 8.23

*ChCl* choline chloride, *BaCl* betaine chloride, *EaCl* ethylamine chloride, *UR* urea, *EG* ethylene glycol, *GLY* glycine, *LAC* lactic acid

**Fig. 1:**
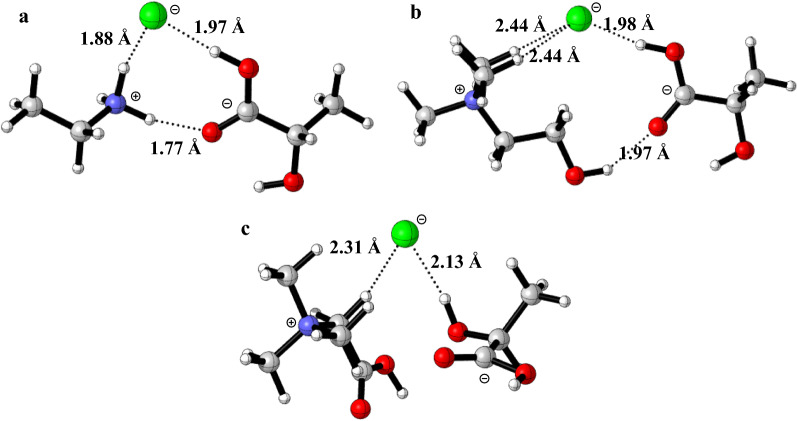
Chemical structures of EaCl:LAC (**a**), ChCl:LAC (**b**) and BaCl:LAC obtained from geometry optimization

The optimized geometries of EaCl:LAC, ChCl:LAC and BaCl:LAC were obtained from DFT calculations. Distance and interactions among HBD and HBA were also analyzed. Three potential hydrogen bonds could formed between EaCl and LAC, which are favorable for the formation of EaCl:LAC (Fig. 1a). In addition, due to the small size of EaCl, the strong electrostatic interaction between nitrogen cation of ethylamine and carboxy group of lactic acid could also contribute to the stabilization of EaCl:LAC. In ChCl:LAC, two hydrogen bonds could be formed between ChCl and LAC (Fig. 1b). However, the distance from nitrogen cation and carboxyl group is too large to form stable interaction. With regard to BaCl:LAC, few interactions were found between BaCl and LAC (Fig. 1c), which might account for the high Δ*G*_rxn_ value and also the difficulties in preparation of BaCl:LAC. It is well known that electrostatic forces of the ions in ILs or DESs could provide or receive H-bond or localize charges on the chemical bonds of the matrix of lignocellulosic biomass. As a result, EaCl:LAC is presumably to display high efficacy in pretreatment of lignocellulosic biomass.

### Evaluation of EaCl:LAC in the pretreatment of lignocellulosic biomass

**Fig. 2:**
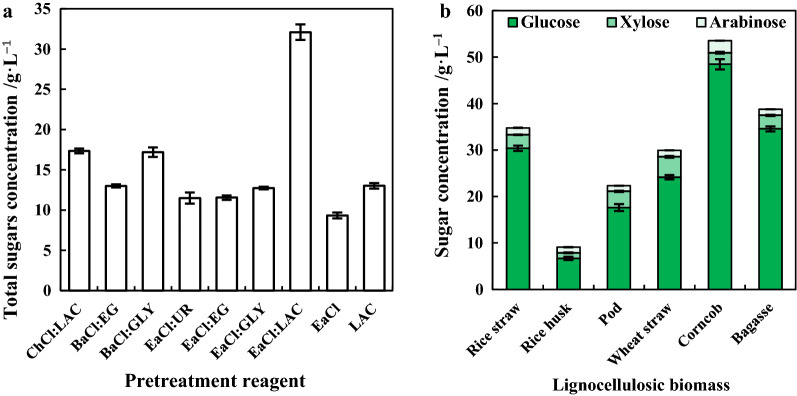
Evaluation of newly synthesized DESs in the pretreatment of various lignocellulosic biomass. **a** Pretreatment of rice straw by various DESs. **b** Pretreatment of various lignocellulosic biomass by EaCl:LAC. ( ), glucose; ( ): xylose, ( ) arabinose

The effect of newly synthesized DESs in pretreatment was investigated with rice straw. ChCl:LAC was regarded as a positive control since it had been applied in pretreatment of rice straw and lignin extraction [39, 40]. Total sugars including glucose, xylose and arabinose were determined. As illustrated in Fig. 2a, EaCl:LAC exhibited the highest efficacy, with total sugars concentration of 32.1 g L^−1^. The concentration of total sugars obtained from ChCl:LAC is 17.4 g L^−1^, ranking the second. The good performance of EaCl:LAC and ChCl:LAC proves the effectiveness of DESs with lactic acid as HBD. About 17.2 g L^−1^ total sugars were achieved for rice straw pretreated by BaCl:GLY, which was similar to that of ChCl:LAC. To further prove the effectiveness of EaCl:LAC, EaCl and LAC were separately applied in the pretreatment of RS. The total sugars concentrations of EaCl and LAC were 9.32 and 13.0 g L^−1^, accounting for 29.0% and 40.6% of that of EaCl:LAC, respectively, indicating the synergistic effect EaCl and LAC. Moreover, the remarkable performance of EaCl:LAC in pretreatment of biomass is in consistence with its structural property.

To further explore the potential of this newly synthesized EaCl:LAC, pretreatment of various lignocellulosic biomass including rice husk, pod, wheat straw, corncob and bagasse were performed. As illustrated in Fig. 2b, EaCl:LAC was effective in the pretreatment of various lignocellulosic biomass except for rice husk. The highest sugar concentration of 53.5 g L^−1^ was obtained with corncob, including 48.5 g L^−1^ glucose, 2.48 g L^−1^ xylose and 2.60 g L^−1^ arabinose. The sugar concentration of corncob was 38.1–489% higher than 38.8 g L^−1^ of bagasse, 34.8 g L^−1^ of rice straw, 29.2 g L^−1^ of wheat straw, 22.3 g L^−1^ of pod and 9.0 g L^−1^ of rice husk, respectively. Furthermore, the total sugars concentration of corncob pretreated by EaCl:LAC was even higher than those of corn stover and RS which were combinatorially pretreated by [Bmim][Cl] and NaOH or ChCl:FA:AA and Na_2_CO_3_ [3, 41]. This newly synthesized ethylamine-based DES, EaCl:LAC, is promising in reducing the recalcitrance of various lignocellulosic biomass.

To further evaluate the effects of EaCl:LAC in reducing the recalcitrance of biomass, component analysis was conducted. Contents of cellulose, hemicellulose and lignin were determined and shown in Table 2. For raw biomass, the cellulose content of corncob was 30.0%, which was higher than that of pod (21.8%) whereas much lower than 38.1% of wheat straw, 35.0% of rice husk, 32.0% bagasse and 31.7% of rice straw. Remarkably, the cellulose content of corncob was increased to 70% after pretreatment with EaCl:LAC. In fact, cellulose contents of all other tested biomass were increased to some extent (11–34%), indicating the effectiveness of EaCl:LAC in reducing recalcitrance of biomass. The cellulose yield of corncob was as high as 98.0%, much higher than other biomass. The hemicellulose contents of corncob, rice straw, pod, wheat straw, bagasse and rice husk were 14.6%, 10.0%, 10.9%, 8.4%, 11.6% and 6.7%, respectively. After EaCl:LAC pretreatment, the hemicellulose removal of 87.9%, 81.1%, 75.3%, 69.9%, 83.1% and 62.7% were achieved for corncob, rice straw, pod, wheat straw, bagasse and rice husk. With regard to lignin including acid-soluble and acid-insoluble, their content in corncob was 26.5%, while in rice straw, pod, wheat straw and rice husk were higher than 30%. The lignin removal of corncob and wheat straw was 71.5% and 67.0%, respectively, much higher than 61.3%, 42.7%, 57.2% and 62.7% of rice straw, pod, bagasse and rice husk. The solid recovery rate of all the tested biomass fell into a range of 40–58%. It should be noted that other components including pigments, proteins, fatty acids, etc., accounted for 9.7–44.5% of raw biomass (Table 2). Most of them could also be efficiently removed by EaCl:LAC pretreatment (Table 2). The results suggest that EaCl:LAC could effectively reduce the stubborn resistance of lignocellulose and lignin in corncob and enhance the cellulose accessibility to cellulase. The excellent performance of EaCl:LAC in lignin removal is consistent with the high potential of DES in the extraction of lignin and metals [32]. In comparison with other DESs in the pretreatment of corncob, EaCl:LAC displayed higher efficiency in removing both hemicellulose and lignin, which is usually difficult to be achieve by choline chloride or betaine-based DESs (Additional file 1) [42]. Generally, combinatorial pretreatment method using alkaline or oxidant should be introduced to achieve higher glucose yield. As a result, the newly developed EaCl:LAC is an efficient pretreatment reagent for lignocellulosic biomass.

**Table 2:** Component analysis of various lignocellulosic biomass before and after treatment with EaCl:LAC

Component (%)	Corncob		Rice straw		Pod		Wheat straw		Bagasse		Rice husk	
	Raw	Treated	Raw	Treated	Raw	Treated	Raw	Treated	Raw	Treated	Raw	Treated
Cellulose	30.0 ± 0.8	70.0 ± 3.1	31.7 ± 2.2	51.0 ± 2.4	21.8 ± 0.1	42.0 ± 0.4	38.1 ± 0.8	63.8 ± 1.4	32.0 ± 1.1	66.0 ± 2.7	35.0 ± 1.7	44.0 ± 1.7
Cellulose yield	‒	98.0	‒	70.7	‒	92.4	‒	92.3	‒	82.5	‒	72.9
Hemicellulose	14.6 ± 0.1	4.2 ± 0.2	10.0 ± 1.2	4.3 ± 0.4	10.9 ± 0.7	5.6 ± 0.7	8.4 ± 0.8	4.6 ± 0.3	11.6 ± 1.4	4.9 ± 0.2	6.7 ± 1.1	4.3 ± 0.4
HC removal^a^	‒	87.9	‒	81.1	‒	75.3	‒	69.9	‒	83.1	‒	62.7
AS lignin^b^	2.3 ± 0.1	0.9 ± 0.1	1.5 ± 0.2	0.7 ± 0.0	1.5 ± 0.2	0.7 ± 0.1	0.9 ± 0.1	0.7 ± 0.0	1.5 ± 0.0	0.9 ± 0.0	0.8 ± 0.1	0.5 ± 0.0
AIS lignin^c^	24.2 ± 1.4	17.1 ± 1.9	31.4 ± 1.2	28.2 ± 0.2	31.1 ± 0.5	38.2 ± 0.5	37.1 ± 1.0	21.9 ± 1.0	9.8 ± 0.6	11.2 ± 1.4	36.1 ± 1.3	29.6 ± 1.2
Lignin removal	‒	71.5	‒	61.3	‒	42.7	‒	67.0	‒	57.2	‒	52.7
Solid yield	‒	42.0	‒	44.0	‒	48.0	‒	55.0	‒	40.0	‒	58.0
Ash	0.4 ± 0.0	0.7 ± 0.0	6.6 ± 0.0	11.7 ± 0.0	0.5 ± 0.1	0.4 ± 0.1	1.0 ± 0.0	1.9 ± 0.0	0.6 ± 0.0	1.2 ± 0.0	11.7 ± 0.2	17.0 ± 1.0
Others	28.9 ± 2.3	7.1 ± 0.7	18.8 ± 3.6	4.1 ± 0.3	34.2 ± 1.1	13.1 ± 0.7	14.5 ± 1.4	7.1 ± 2.0	44.5 ± 3.0	18.8 ± 1.9	9.7 ± 1.0	4.6 ± 1.7

^a^HC removal: hemicellulose removal

^b^AS lignin: acid-soluble lignin

^c^AIS lignin: acid-insoluble lignin

### Physical characterization of corncob pretreated by EaCl:LAC

In corncob, lignin and hemicellulose form a tight network structure wrapping around the outer layer of cellulose, which seriously hinders the accessibility of cellulose by cellulase [13]. SEM analysis was implemented to monitor the surface structure of untreated and pretreated corncobs (Additional file 2). In untreated corncob, a smooth and compact surface with strong rigid structure was observed. However, an entirely different landscape was detected in the pretreated corncob. The surface of pretreated corncob became loose and rough with obvious fracture delamination, revealing destroyed lignin and hemicellulose around cellulose, which was favorable for improved cellulose accessibility in corncob. Moreover, the observed changes in corncob surface are consistent with the high lignin and hemicellulose removal after EaCl:LAC pretreatment.

Furthermore, XRD assay was conducted to explore changes of the crystallinity index (CrI) of untreated and pretreated corncobs. According to the overlapped XRD spectrum (Additional file 3), no new peak appeared in the pretreated corncob, indicating no structural change after pretreatment. The diffraction peaks at 16° and 21° represent the typical crystalline structures of cellulose I, and could be used to calculate CrI [11]. The CrI value could be regarded as an indicator for the exposure and accessibility of cellulose. Above two characteristic absorption peaks of pretreated corncob were much higher than those of raw corncob, largely due to the increased cellulose content after removal of lignin and hemicellulose. The CrI values of raw and pretreated corncob were 31.0% and 42.8%, respectively. The increased CrI value of pretreated corncob agrees with the increased cellulose content (Table 2), and indicates the successful removal of certain stubborn components wrapped around the cellulose. It is favorable for increasing the accessibility of cellulase to cellulose in lignocellulosic biomass [43].

FTIR spectrum of untreated and pretreated corncobs was obtained (Additional file 4). The absorption peaks at 830 and 1166 cm^−1^ refer to the vibration of C–C bond in lignin, indicating the lignin in corncob is SGH lignin (syringyl-guaiacyl-*p*-hydroxyphenyl) [44]. In comparison with untreated corncob, the characteristic absorption peaks of lignin in pretreated corncob were significantly reduced, revealing that a large amount of lignin was removed. The absorption peak at 1638 cm^−1^ is attributed to the stretching vibration of γ-lactone, and the decrease value means that the lignin was largely removed after pretreatment [28, 45]. The increased absorption peak at 895 cm^−1^, relating to *β*-glycosidic bond in cellulose, indicates the removal of hemicellulose and exposure of more cellulose. Furthermore, the absorption peak at 1383 cm^−1^ is caused by the stretching vibration of C–H bond in cellulose, and the increased value shows that the amorphous cellulose was removed after EaCl:LAC pretreatment. The absorption peak at 1736 cm^−1^ represents the vibration of carboxyl group in hemicellulose, and the decreased adsorption peak of pretreated corncob reveals the removal of hemicellulose in comparison with raw corncob [44]. In summary, the FTIR result agrees with the composition analysis. After pretreatment with EaCl:LAC, a large amount of lignin and hemicellulose in corncob were removed, and the relative content of cellulose was significantly increased to 70.0%, resulting in enhanced cellulose accessibility.

### Development of fed-batch pretreatment process

To establish an efficient and economic corncob pretreatment process, various factors were optimized. Firstly, conditions including temperature, incubation time and solid–liquid ratios were systematically investigated, and the resultant corncobs pretreated by EaCl:LAC were subjected to enzymatic hydrolysis for determination of total sugars (Additional file 5). At 90 °C and 110 °C, elongated pretreatment time from 0.5 to 3.0 h resulted in higher total sugars. However, when the temperature increased to 130 °C and 150 °C, different profiles were observed. At over 130 ℃, longer incubation time led to decreased total sugars, which might be attributed to the destruction of cellulose structure. As a result, either high temperature for short time or low temperature for long time is beneficial to the performance of EaCl:LAC. Under the optimum pretreatment conditions of 150 °C for 0.5 h and solid–liquid ratio of 1:15, the highest total sugars concentration of about 55.6 g L^−1^ were obtained from the pretreated corncob (Additional file 5).

**Fig. 3:**
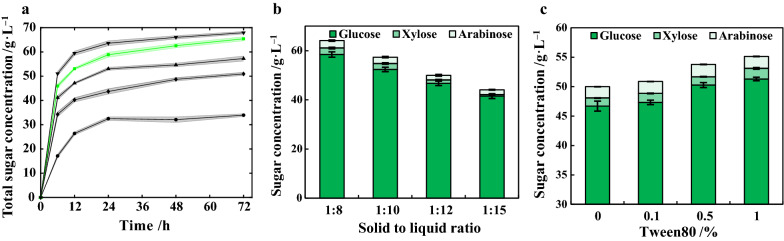
Optimization of enzymatic hydrolysis conditions. **a** Cellulase loading and hydrolysis time, ( ): 70 FPU g^−1^; ( ): 50 FPU g^−1^, ( ) 40 FPU g^−1^, ( ) 30 FPU g^−1^, ( ) 10 FPU g^−1^, shadow refers to standard deviation. **b** Solid–liquid ratio, ( ): glucose; ( ): xylose, ( ): arabinose. **c** Tween80, ( ): glucose; ( ): xylose, ( ): arabinose. All pretreatment was performed in triplicate

Furthermore, factors including cellulase dosage, hydrolysis time, solid to liquid ratio and supplementation of Tween80, which might influence the enzymatic hydrolysis process, were investigated. Different amounts of cellulase, ranging from 10 to 70 FPU g^−1^ pretreated corncob, were loaded, and the released total sugars were monitored as illustrated in Fig. 3a. Along with the increase of hydrolysis time, the total sugars elevated rapidly during the initial 24 h, and then slowly increased until 72 h. Although longer hydrolysis time could lead to higher concentrations of total sugars, it also results in compromised space–time yield. At 50 FPU g^−1^ cellulase, total sugars of 57.0 g L^−1^ was obtained at 24 h, merely 4.5 g L^−1^ lower than that of 70 FPU g^−1^ cellulase. Considering the relative lower loading of cellulase and higher efficiency, hydrolysis with 50 FPU g^−1^ cellulase for 24 h was selected as the suitable condition. Influence of solid to liquid ratios at 1:8, 1:10, 1:12 and 1:15 on releasing of total sugars were also investigated at 50 FPU g^−1^ cellulase (Fig. 3b). Increased liquid ratios represent lower addition of biomass. Along with the increase of solid to liquid ratios, the total sugars decreased from 64 to 44 g L^−1^ after 24 h of hydrolysis. The total sugar yield per pretreated corncob increased from 513 to 661 g kg^−1^. Although enhanced total sugar yield could be achieved at a relatively high liquid ratio at the expense of cellulase, however, lower titer of total sugars could be resulted, which is disadvantageous for biobutanol fermentation since extra energy-consuming concentration step is required to increase the sugar concentration. In the view of better mass transferring and relatively higher total sugars, solid to liquid ratio of 1:12 is considered as optimum, at which the total sugars of 50 g L^−1^ could be achieved after 24 h of hydrolysis.

Although most of the lignin and hemicellulose could be removed from corncob after EaCl:LAC pretreatment, residual lignin could competitively adsorb free cellulase, which might result in losing of cellulase and impairing hydrolysis efficiency. Supplementation of bovine serum albumin (BSA) or Tween80 has been proved to be effective solutions for reducing inefficient adsorption of cellulase on lignin and deactivation of absorbed cellulase by enzyme–substrate interaction [46, 47]. Herein, addition of Tween80 was also attempted (Fig. 3c). In comparison with the control (without Tween80), supplementation of 0.1–1.0% (v/v) of Tween80 resulted in increased total sugars. At 1.0% Tween80, total sugars concentration of as high as 55.1 g L^−1^ was attained, 10.2% higher than 50.0 g L^−1^ of control. Excessive addition of Tween80 could however complicate the compositions and affect the biocompatibility of hydrolysates in biobutanol fermentation. At 0.5% Tween80, the total sugar reached 53.8 g L^−1^, which was adequate for butanol fermentation [38]. As a result, addition of 0.5% Tween80 is selected for the hydrolysis of pretreated corncob into fermentable sugars.

**Fig. 4:**
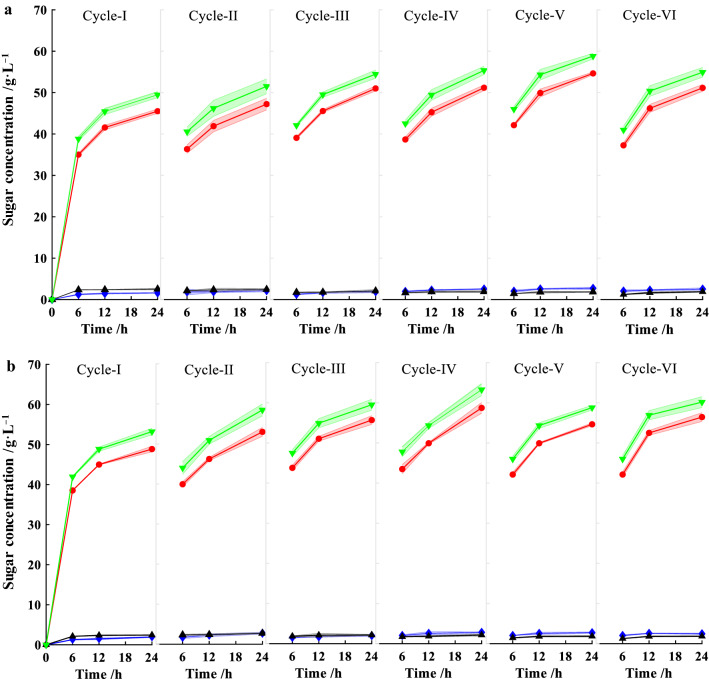
Reusability of cellulase absorbed on pretreated corncob. **a** Without addition of Tween80, **b** 0.5% (v/v) Tween80. ( ): total sugar; ( ): glucose; ( ): xylose; ( ): arabinose. Shadow refers to standard deviation, and all reactions were performed in triplicate

To further reduce the enzyme loading, cellulases absorbed on residual corncobs were recycled and reused in the consecutive batches. Herein, two processes with and without addition of 0.5% Tween80 were evaluated. At the end of each batch, the residual solids which might absorb cellulases as previous reported [3], were collected and reloaded into the next batch. The loadings of cellulase were decreased by 5 FPU g^−1^ for the following batches. The absorbed cellulases were recycled for five times, and sugars including glucose, xylose and arabinose were determined and illustrated in Fig. 4. The total sugars increased rapidly within the initial 6 h, and the addition of cellulase attached to corncob did not result in a decrease in enzymatic efficiency since it could lead to compromised mass transfer compared with the first batch (Cycle I). In the process with Tween80 (Fig. 4b), total sugars concentration of Cycle I was 52.9 g L^–1^, while it was 49.5 g L^−1^ in control (without Tween80) (Fig. 4a). Addition of Tween80 was favorable for the enzymatic hydrolysis, exhibiting 7–14% increase in total sugars at each batch. In the sixth batch (Cycle IV), only 25 FPU g^−1^ of fresh cellulase was supplemented. The total sugars reached 58.8 g L^−1^ and 54.9 g L^−1^ for processes with and without Tween80, respectively, which were 706 and 659 g kg^−1^ corncob pretreated by EaCl:LAC. The total sugars increased by about 11% than that of Cycle I. It is presumed that Tween80 might reduce the inactivation of cellulase caused by interaction between cellulase and substrate. Thus, the cellulases adsorbed on corncob displayed stable and even improved enzymatic activity in the next cycle, which is consistent with previous study [47]. The total sugars concentrations of each batch were enough as carbon source for the butanol fermentation with *C. saccharobutylicum*. It should be noted that about 50% of cellulases could be saved through this newly developed recycling process.

### Biobutanol fermentation with corncob hydrolysates by *C. saccharobutylicum* DSM13864

**Fig. 5:**
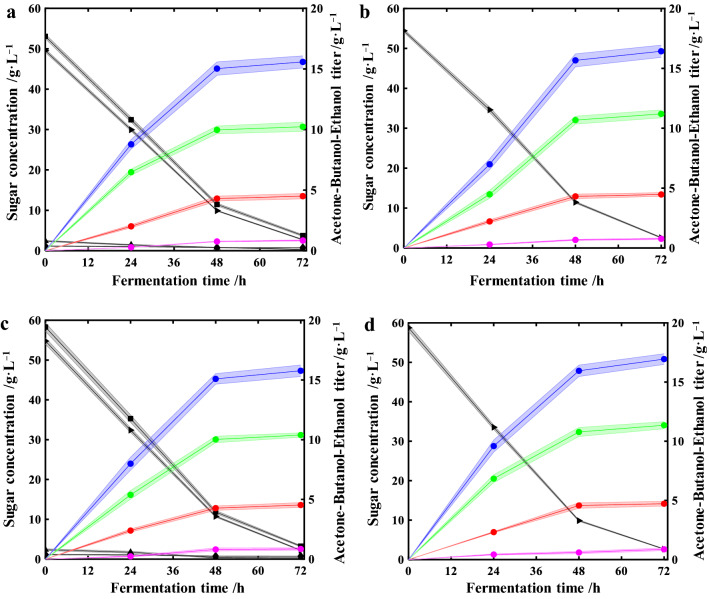
Biobutanol production from hydrolysates of corncob and glucose as carbon sources. **a** Hydrolysate of Cycle VI, **b** control I (54 g L^−1^ glucose), **c** hydrolysate of Cycle VI_Tween80,_ **d** control II (59 g L^−1^ glucose). ( ): total sugars; ( ): glucose; ( ): xylose; ( ): arabinose; ( ): total solvents; ( ): butanol; ( ): ethanol; ( ): acetone. Shadow refers to standard deviation, and all fermentations were performed in triplicate

Application of hydrolysates from EaCl:LAC-pretreated corncob was evaluated in biobutanol fermentation. *C. saccharobutylicum* DSM13864 could utilize pentoses, such as xylose, as carbon sources and is regarded as one of the most promising bacteria for biobutanol fermentation. Hydrolysates of the sixth batch were collected and designated as Cycle VI_Tween80_ and Cycle VI for with and without Tween80 addition, respectively. The total sugars concentrations of Cycle VI_Tweeen80_ and Cycle VI were determined to be 58.8 g L^−1^ and 54.9 g L^−1^. Control experiments were also carried out with glucose as carbon source instead of hydrolysates. The glucose concentrations of the control groups were kept at the same level with the total sugars of the hydrolysates from Cycle VI_Tween80_ and Cycle VI. Consumption of reducing sugars and production of acetone, butanol and ethanol (ABE) were monitored and illustrated in Fig. 5 and Table 3. During the initial 48 h, *C. saccharobutylicum* grew quickly with high sugar consumption and ABE production rates. After 48 h, ABE production decreased, along with a slower sugar consumption rate. After 72 h, butanol titers of 10.2 and 10.4 g L^−1^ were reached for Cycle VI (Fig. 5a) and Cycle VI_Tween80_ (Fig. 5c), respectively, slightly lower than the corresponding glucose controls of 11.2 (Fig. 5b) and 11.4 g L^−1^ (Fig. 5d). Although the concentrations of total sugars in hydrolysates are at same level with glucose controls, they are mixture of arabinose, xylose and glucose. The lower ABE titers of hydrolysates might be attributed to the lower glucose concentrations and the complicated metabolite fluxes of hydrolysates. It should be noted that the butanol yield and productivity of Cycle VI_Tween80_ and Cycle VI hydrolysates were 194 ${\text{g}}\;{\text{kg}}_{{{\text{sugar}}}}^{{ - 1\;{\text{total}}}}$ and 0.15 g L^−1^ h^−1^, and 206 ${\text{g}}\;{\text{kg}}_{{{\text{sugar}}}}^{{ - 1\;{\text{total}}}}$, and 0.14 g L^−1^ h^−1^, respectively, which are at similar level with those of glucose controls (Table 3). The specific yields of butanol of Cycle VI_Tween80_ and Cycle VI per pretreated corncob were 137 and 136 g kg^−1^. With regard to total solvents of ABE, the titers of Cycle VI_Tween80_ and Cycle VI were 15.8 and 15.6 g L^−1^, with calculated yields per total sugars of 295 and 315 ${\text{g}}\;{\text{kg}}_{{{\text{sugar}}}}^{{ - 1\;{\text{total}}}}$, and calculated yields per pretreated corncob of 208 and 207 g kg^−1^, respectively. As a result, corncob hydrolysates from Cycle VI could be efficiently utilized by *C. saccharobutylicum* as carbon source for biobutanol fermentation. Moreover, the corncob hydrolysates did not display obvious inhibitory effect on the growth and biobutanol production of *C. saccharobutylicum*.

**Table 3:** Biobutanol fermentation with corncob hydrolysates by *C. saccharobutylicum* DSM13864

Carbon source	Butanol			Acetone–butanol–ethanol (ABE)		
	Titer (g L^−1^)	Yield^a^ (g L^−1^)	Prod.^b^ (g L^−1^ h^−1^)	Titer (g L^−1^)	Yield^a^ (g kg^−1^)	Prod. (g L^−1^ h^−1^)
Cycle VI	10.2	206 (136)	0.14	15.6	315 (207)	0.21
Control I^c^	11.2	210	0.15	16.3	320	0.22
Cycle VI_Tween80_	10.4	194 (137)	0.15	15.8	295 (208)	0.22
Control II^d^	11.4	206	0.16	16.9	306	0.23

^a^Numbers outside the brackets represent specific yields per total sugars or glucose, numbers inside the brackets refer to specific yields per pretreated corncob

^b^Prod.: productivity

^c^Control I: 54 g L^−1^ glucose

^d^Control II: 59 g L^−1^ glucose

This study provides a simple and biocompatible process for the facile conversion of corncob into biobutanol. Compared with other established processes, EaCl:LAC is a low-price, environmental friendly and biocompatible reagent. The specific ABE yields per pretreated and raw biomass of this process were calculated to be 208 and 87.4 g kg^−1^, second only to that of corncob pretreated by 0.5 M NaOH [48]. In view of its low price and high biocompatibility, EaCl:LAC is more efficient and promising than traditional ionic liquids such as [Bmim][Cl], and DESs such as ChCl:FA and ChCl:FA:AA. There is no need to add other reagents which are commonly used in combinatorial pretreatments, such as NaOH or Na_2_CO_3_ [3, 41]. Moreover, EaCl:LAC could also be facilely recycled by filtration. The hydrolysates with high titers of reducing sugars could presumably be applied in the biosynthesis of other biofuels, amino acids and high-valued natural products. Consequently, this study provides a promising reagent for significantly reducing the recalcitrance of lignocellulosic biomass, and an economic cellulase recycling process for biobutanol production.

## Conclusions

In this study, several DESs, based on betaine and ethylamine as hydrogen bond acceptors, were newly synthesized and evaluated in the pretreatment of various lignocellulosic biomass. EaCl:LAC with lactic acid as hydrogen bond donor was the most efficient for reducing the recalcitrance of lignocellulosic biomass. Only employing single pretreatment with EaCl:LAC, both high hemicellulose and lignin removal were achieved. Facile pretreatment process was established with recycled cellulase. The hydrolysate of pretreated corncob was biocompatible and could be directly utilized by *C. saccharobutylicum* for biobutanol fermentation with similar butanol titer and yield as glucose counterpart.

## Methods

### Biomass, chemicals and strains

All lignocellulosic biomass used in this study was sourced from a farm in Jinan, Shandong province, China. The biomass was milled by grinder and passed through a 380-μm sieve, and dried at 60 °C for 24 h before use.

Ethylamine chloride (EaCl) was purchased from Macklin Biochemical Co., Ltd. Cellulase was a generous gift from *Vland* Biotech Co Ltd. All other chemicals were of analytical grade and purchased from Sinopharm Chemical Reagent Co., Ltd.

*Clostridium saccharobutylicum* DSM 13864 was purchased from DSMZ. In order to induce sporulation, it was cultivated in Reinforced Clostridia Medium (RCM) at 37 °C for 7 days and stored at room temperature. Spore suspension (10%, v/v) was inoculated in 12 mL sterilized RCM and transferred to a desiccator evacuated to a vacuum level of 0.065 MPa. Afterwards, the culture was cultivated at 37 °C for 12–18 h for further used as the seed medium [27].

### Synthesis of ethylamine chloride-based DES

ChCl, BaCl and EaCl as hydrogen bond acceptor, and lactic acid, ethyl glycol, glycerol and urea as hydrogen bond donors were mixed at the ratios as listed in Table 1. The mixture was heated and stirred at 180 rpm in a conical flask with plug to reduce volatilization until a homogenous colorless liquid was formed. The DESs were kept in vacuum desiccator with silica gel for further use.

### DFT calculations

The initial structures for these DESs were constructed with ChemDraw software and optimized via DFT calculations using Gaussian 09 suite. Functionals of B3LYP [49], M062X [37], ωB97XD [50] and basis set of 6 − 311 + G** were selected to optimize the geometrical structures and calculate the free energy differences. Grimme correction was performed for entropy and Head-Gordon correction was performed for enthalpy. The coordinates of the optimized structure of DESs could be found in Additional file 6.

### Pretreatment of corncob with EaCl:LAC

Ten grams of corncob was added into a three-necked flask containing 150 g of EaCl:LAC, followed by heating up to 150 °C in an oil bath. Then the mixture was incubated for 0.5 h with mechanical agitation (200 rpm). Furthermore, cellulose was regenerated by adding appropriate volume of hot deionized water (85 °C). The regenerated cellulose was filtrated with a 380-mesh sieve, and then washed with water and dehydrated to obtain the pretreated corncob, which was stored at 4 °C for further use.

Effects of temperature, pretreatment time and solid to liquid ratio on the pretreatment of corncob with EaCl:LAC were performed as mentioned above, except for varying the incubation temperature of 90, 110, 130 and 150 °C, pretreatment time of 0.5, 1.0, 1.5 and 2.0 h, solid to liquid ratios of 1:8, 1:10, 1:12 and 1:15. The pretreated corncobs were subjected to hydrolysis with cellulase and the released sugars were determined employing HPLC (Agilent 1100) equipped with an Aminex HPX-87H column at 60 °C with 5 mM H_2_SO_4_ as eluent at a flow rate of 0.6 mL min^−1^ [41].

### Enzymatic hydrolysis of pretreated corncob

One gram of the pretreated corncob was added to 12 mL citrate buffer (50 mM, pH 4.8) containing 100 μL ampicillin (1 g L^−1^) and 50 FPU cellulase in a 50-mL flask. The mixture was incubated in a water bath at 50 °C and 120 rpm for 24 h for releasing of arabinose, xylose and glucose. Samples (300 μL) were withdrawn at 6, 12, 24 h and centrifuged at 12,000×*g* for 10 min. The resultant supernatants (100 μL) were mixed with 900 μL ultrapure water. The concentrations of arabinose, xylose and glucose were determined as above described.

Effect of cellulase dosage, hydrolysis time, solid to liquid ratios were investigated. Cellulase was supplemented at dosages of 10, 30, 40, 60 and 70 FPU g^−1^ pretreated corncob. Different solid to liquid ratios of 1:8, 1:10, 1:12 and 1:15 were adopted. Tween80 was also added in the hydrolysis mixture at 0.1%, 0.5% and 1.0%. Samples (100 μL) were withdrawn from the reaction mixture at 24, 48, and 72 h, and then analyzed as above mentioned.

### Recovery of cellulases adsorbed to corncob

Cycle I of enzymatic hydrolysis was conducted in a 250-mL conical flask, consisted of 5 g pretreated corncob dispersing in 60 mL citrate buffer (50 mM, pH 4.8) and 50 FPU cellulase. After 24 h, the cellulase adsorbed on residual corncob was collected by filtration and supplemented to the next cycle. Based on previous study, the amount of cellulase added could be reduced by 10% for each cycle to achieve similar level of total sugars as Cycle I. Samples were prepared and analyzed as above mentioned.

### Biobutanol fermentation of *C. saccharobutylicum* DSM 13864

The corncob hydrolysates of Cycle VI_Tween80_ and Cycle VI was utilized as carbon source for butanol fermentation by *C. saccharobutylicum* DSM 13864. Other components of fermentation medium included 10 g L^−1^ of corn steep liquor (CSL), 4 g L^−1^ of CaCO_3_, 2 g L^−1^ of (NH_4_)_2_SO_4_, 0.5 g L^−1^ of K_2_HPO_4_ and 0.01 g L^−1^ of MnSO_4_·H_2_O. Furthermore, the pH of medium was adjusted to 6.5 with 4.0 M NaOH and autoclaved at 115 °C for 20 min. Control experiment was conducted with fermentation medium containing 54 or 59 g L^−1^ of glucose. 10% (v/v) of actively growing cell culture was inoculated into sterilized fermentation medium, and anaerobically incubated at 37 °C in a desiccator (0.065 MPa) [4]. Samples were withdrawn at different time intervals and the contents of acetone, butanol and ethanol (ABE) were analyzed by GC according to previously described methods [27]. All fermentation experiments were carried out in triplicate.

### Component analysis and physical characterization of corncob pretreated by EaCl:LAC

#### Component analysis

Amount of cellulose, hemicellulose, lignin and ash in raw and pretreated corncobs was determined according to previously reported methods [4]. Removal of hemicellulose and lignin was calculated according to the following formulas:

$${\text{Hemicellulose}}\;{\text{removal}}\;(\% ) = \left( {1 - \frac{{{\text{Hemicellulose}}\;{\text{in}}\;{\text{pretreated}}\;{\text{corncob}}}}{{{\text{Hemicellulose}}\;{\text{in}}\;{\text{untreated}}\;{\text{corncob}}}} \times {\text{solid}}\;{\text{yield}}} \right) \times 100\% ;$$

$${\text{Delignification}}\;(\% ) = \left( {1 - \frac{{{\text{Lignin}}\;{\text{in}}\;{\text{pretreated}}\;{\text{corncob}}}}{{{\text{Lignin}}\;{\text{in}}\;{\text{untreated}}\;{\text{corncob}}}} \times {\text{solid}}\;{\text{yield}}} \right) \times 100\% .$$

#### SEM analysis

Scanning electron microscopy (5.0 kV, 1200× Hitachi S-4800, Japan) analysis was operated to monitor the surface morphological features of corncob before and after pretreatment.

#### XRD analysis

The crystallinity of corncob was measured with X-ray diffractometer (XRD), using a D/max 2500 PC diffractometer with Cu/Ka radiation source (Rigaku Corporation, Tokyo, Japan). It was operated at a voltage of 60 kV and a current of 300 Ma with a scanning speed of 0.02°/min and the 2*θ* range from 5° to 40°. Crystallinity index (CrI) was calculated as following.

$${\text{CrI }}(\% ) = \frac{{I_{{002}} - I_{{{\text{am}}}} }}{{I_{{002}} }} \times 100\% .$$

*I*_002_ and *I*_am_ imply the intensities of the peaks at near 21.4° and 16.0°, respectively.

#### FTIR analysis

FTIR was performed to detect the chemical structure of corncob using a Nicolet PROTÉGÉ 460 FTIR Spectrometer (Nicolet, Thermo Scientific, Shanghai, People’s Republic of China) [20]. FTIR spectra of the samples was recorded between 2000 and 600 cm^−1^.

## Supplementary information

**Additional file 1.** Comparison on pretreatment of corncob using different DESs.

**Additional file 2.** SEM analysis of raw or pretreated corncob.

**Additional file 3.** XRD data of raw and pretreated corncob.

**Additional file 4.** FTIR spectra of raw and pretreated corncob.

**Additional file 5.** Optimization the pretreatment conditions of corncob using EaCl:LAC.

**Additional file 6.** Coordinates of the optimized structure of DESs.

## Data Availability

The datasets generated during this study are included in this published article and its Additional files.

## Abbreviations

DES: Deep eutectic solvent
BaCl: Betaine chloride
EaCl: Ethylamine chloride
ChCl: Choline chloride
LAC: Lactic acid
EG: Ethyl glycol
GLY: Glycerol
UR: Urea
DFT: Density functional theory
CrI: Crystallinity index
BSA: Bovine serum albumin
Cycle I: Hydrolysate of the first batch
Cycle I_Tween80_: Hydrolysate of the first batch with addition of Tween80
Cycle VI: Hydrolysate of the sixth batch
Cycle IV_Tween80_: Hydrolysate of the sixth batch with addition of Tween80
Control I: Control medium with 54 g L^−^^1^ glucose
Control II: Control medium with 59 g L^−^^1^ glucose
ABE: Acetone, butanol and ethanol
