# Supplementary material for: Facilely reducing recalcitrance of lignocellulosic biomass by a newly developed ethylamine-based deep eutectic solvent for biobutanol fermentation
Source: Biotechnol Biofuels. 2020 Oct 9;13:166. doi: 10.1186/s13068-020-01806-9 (PMC7547450; doi:10.1186/s13068-020-01806-9)
Supplement: Supplementary file 1 — Additional file 1. Comparison on pretreatment of corncob using different DESs. [file 13068_2020_1806_MOESM1_ESM.docx]

**Table S1** Comparison on pretreatment of corncob using different deep eutectic solvents

| DES | Hemicellulose removal  [%] | Lignin removal  [%] | Total sugars concentration/Glucose yield  [g/L]/[%] | Ref. |
| --- | --- | --- | --- | --- |
| Ch-Cl:Urea | *n. a.^a^* | 88 | 16/76 | Procentese et al., 2015 |
| Glycerol:ChCl | *n. a.* | 71.3 | –/96.4 | Zhang et al., 2016 |
| BTMAC:LA | 80.8 | 63.4 | –/94.0 | Guo et al., 2019 |
| [Bet][AA] | 47.7 | 49.1 | –/58 | Liang et al., 2020 |
| EaCl:LAC | 87.9 | 71.5 | 64/85.5 | This study |

*^a^* *n. a.*: not available.

[1] Procentese A, Johnson E, Orr V, Campanile AG, Wood JA, Marzocchella A, Rehmann L. Deep eutectic solvent pretreatment and subsequent saccharification of corncob. Bioresour. Technol., 2015;192:31-36.

[2] Zhang CW, Xia SQ, Ma PS. Facile pretreatment of lignocellulosic biomass using deep eutectic solvents. Bioresour. Technol., 2016;219:1-9.

[3] Guo W, Zhang QL, You TT, Zhang X, Xu F, Xu YY, Short-time deep eutectic solvent pretreatment for enhanced enzymatic saccharification and lignin valorization. Green Chem., 2019:21:3099-3108.

[4] Liang Y, Duan WJ, An XX, Qiao YY, Tian YY, Zhou HF. Novel betaine-amine acid based natural deep eutectic solvents for enhancing the enzymatic hydrolysis of corncob. Bioresource Technol., 2020;310:123389.
