# Supplementary material for: Facilely reducing recalcitrance of lignocellulosic biomass by a newly developed ethylamine-based deep eutectic solvent for biobutanol fermentation
Source: Biotechnol Biofuels. 2020 Oct 9;13:166. doi: 10.1186/s13068-020-01806-9 (PMC7547450; doi:10.1186/s13068-020-01806-9)
Supplement: Supplementary file 2 — Additional file 2. SEM analysis of raw or pretreated corncob. [file 13068_2020_1806_MOESM2_ESM.pptx]

## Slide 1
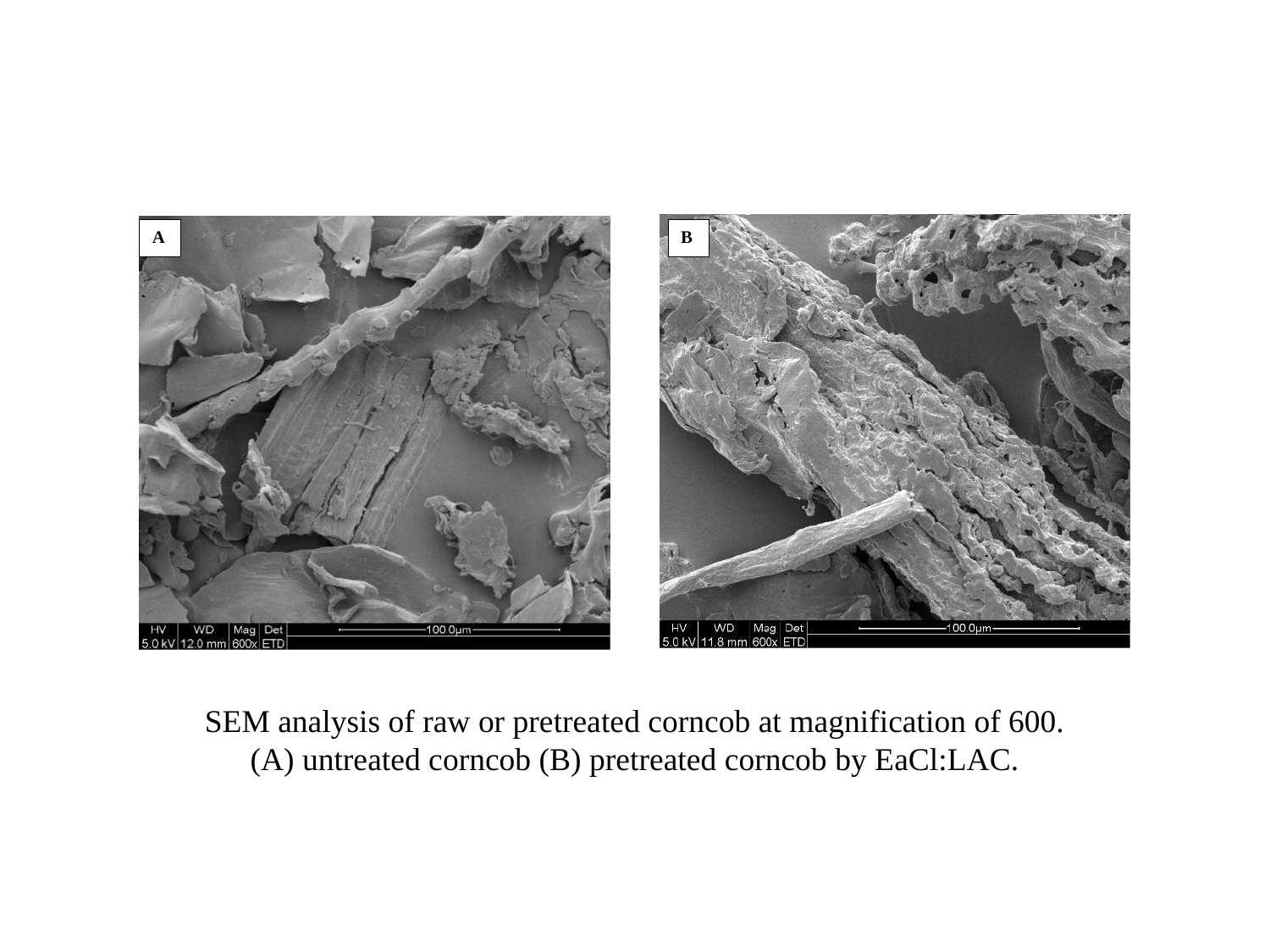

A
B
SEM analysis of raw or pretreated corncob at magnification of 600.
(A) untreated corncob (B) pretreated corncob by EaCl:LAC.
