# Supplementary figures and images for: Facilely reducing recalcitrance of lignocellulosic biomass by a newly developed ethylamine-based deep eutectic solvent for biobutanol fermentation
Source: Biotechnol Biofuels. 2020 Oct 9;13:166. doi: 10.1186/s13068-020-01806-9 (PMC7547450; doi:10.1186/s13068-020-01806-9)

## Slide 1
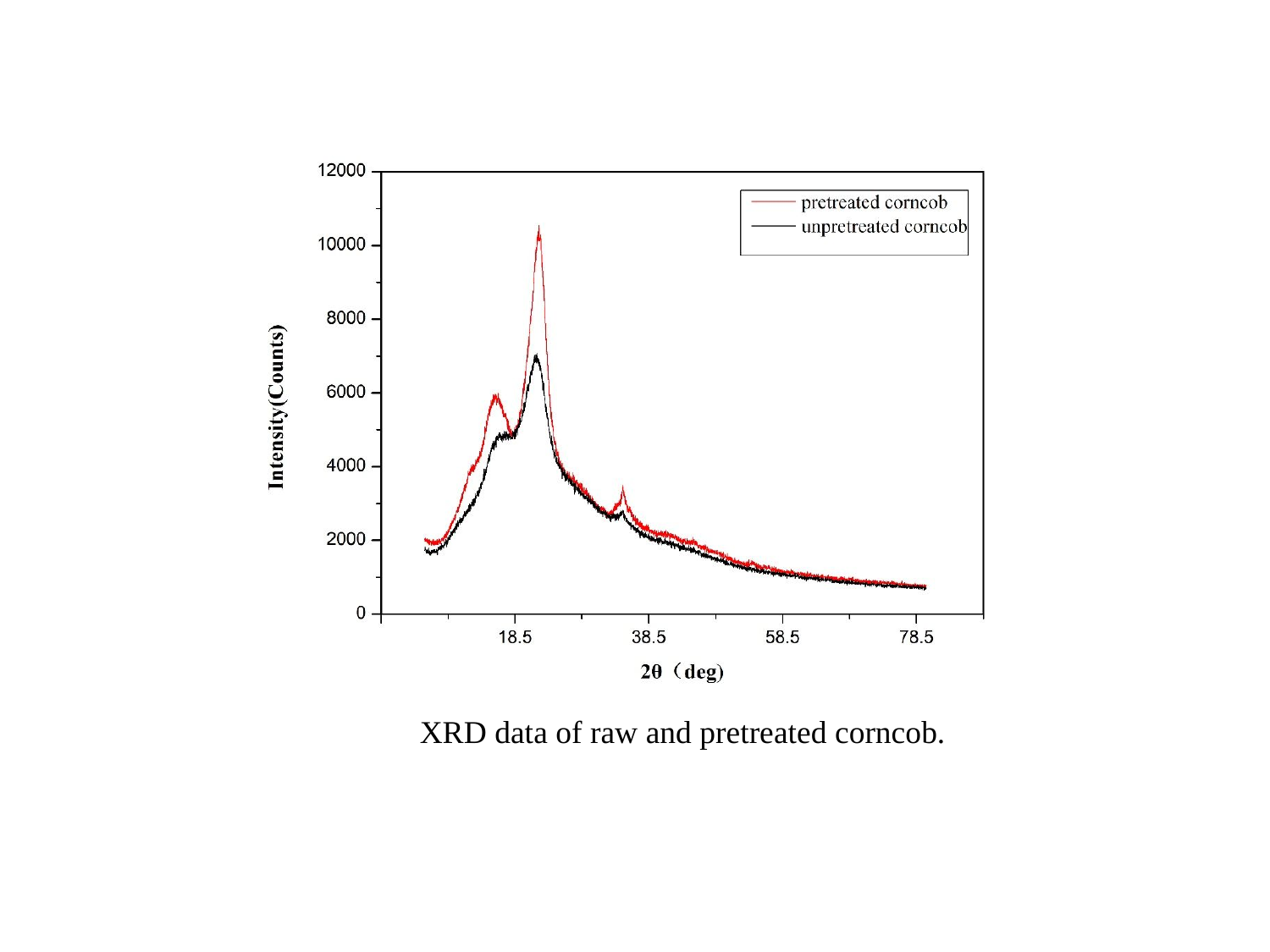

XRD data of raw and pretreated corncob.

Supplement: Supplementary file 3 — Additional file 3. XRD data of raw and pretreated corncob. [file 13068_2020_1806_MOESM3_ESM.pptx]

## Slide 1
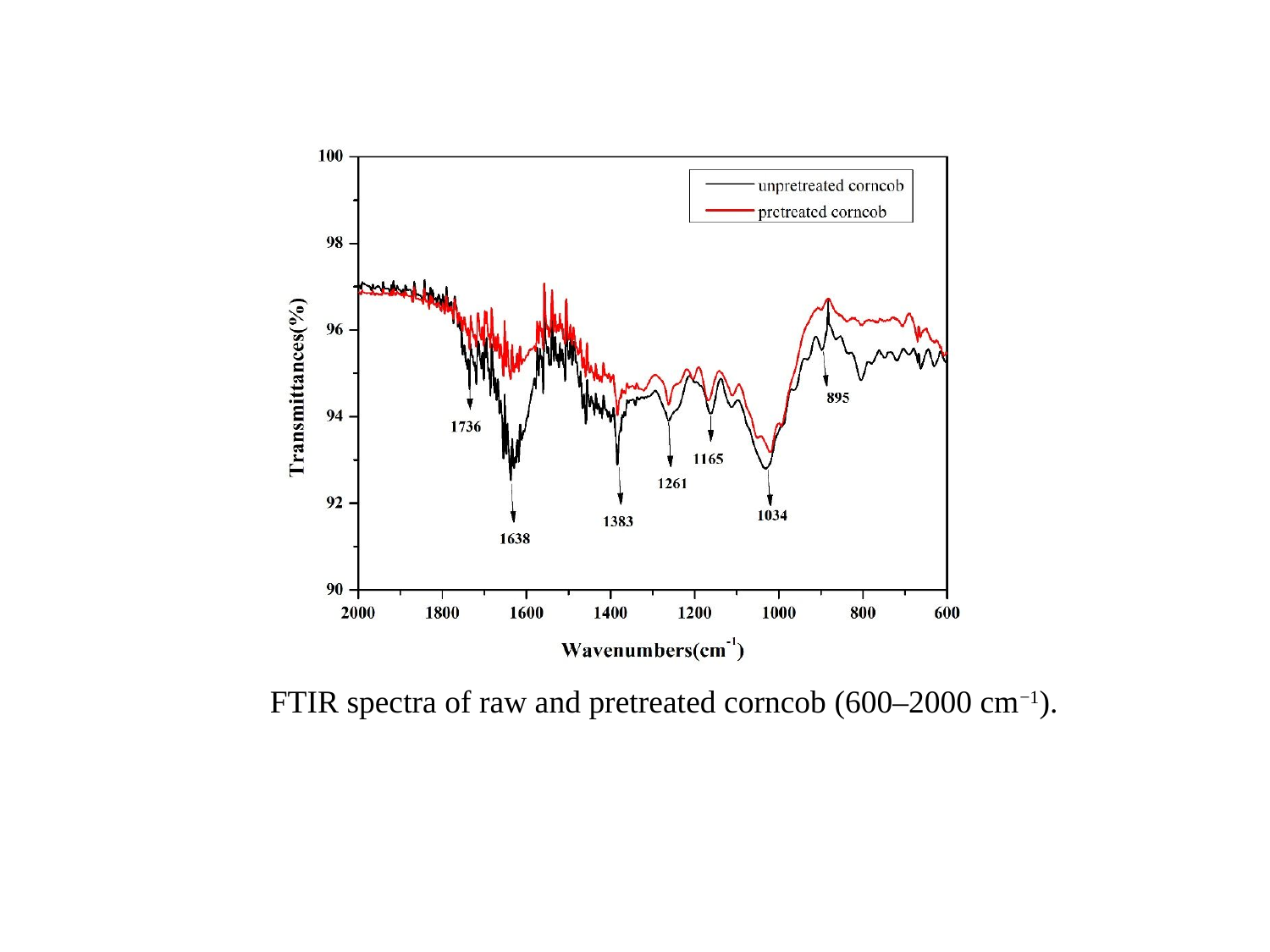

FTIR spectra of raw and pretreated corncob (600–2000 cm−1).

Supplement: Supplementary file 4 — Additional file 4. FTIR spectra of raw and pretreated corncob. [file 13068_2020_1806_MOESM4_ESM.pptx]
