# Supplementary material for: Facilely reducing recalcitrance of lignocellulosic biomass by a newly developed ethylamine-based deep eutectic solvent for biobutanol fermentation
Source: Biotechnol Biofuels. 2020 Oct 9;13:166. doi: 10.1186/s13068-020-01806-9 (PMC7547450; doi:10.1186/s13068-020-01806-9)
Supplement: Supplementary file 5 — Additional file 5. Optimization the pretreatment conditions of corncob using EaCl:LAC. [file 13068_2020_1806_MOESM5_ESM.docx]

**Table S2** Optimization the pretreatment conditions of corncob using EaCl:LAC

| **Entry** | **Temperature/℃** | **Time/h** | **Solid-Liquid ratio** | **Total sugar**  **(g·L^-1^)** |
| --- | --- | --- | --- | --- |
| 1 | 90 | 0.5 | 1:8 | 12.97 |
| 2 | 90 | 1 | 1:10 | 21.42 |
| 3 | 90 | 1.5 | 1:12 | 27.12 |
| 4 | 90 | 2 | 1:15 | 33.95 |
| 5 | 110 | 0.5 | 1:10 | 30.13 |
| 6 | 110 | 1 | 1:8 | 41.17 |
| 7 | 110 | 1.5 | 1:15 | 42.68 |
| 8 | 110 | 2 | 1:12 | 47.04 |
| 9 | 130 | 0.5 | 1:12 | 45.76 |
| 10 | 130 | 1 | 1:15 | 49.12 |
| 11 | 130 | 1.5 | 1:8 | 40.86 |
| 12 | 130 | 2 | 1:10 | 41.69 |
| **13** | **150** | **0.5** | **1:15** | **55.60** |
| 14 | 150 | 1 | 1:12 | 44.23 |
| 15 | 150 | 1.5 | 1:10 | 28.17 |
| 16 | 150 | 2 | 1:8 | 26.28 |
