# Supplementary material for: Facilely reducing recalcitrance of lignocellulosic biomass by a newly developed ethylamine-based deep eutectic solvent for biobutanol fermentation
Source: Biotechnol Biofuels. 2020 Oct 9;13:166. doi: 10.1186/s13068-020-01806-9 (PMC7547450; doi:10.1186/s13068-020-01806-9)
Supplement: Supplementary file 6 — Additional file 6. Coordinates of the optimized structure of DESs. [file 13068_2020_1806_MOESM6_ESM.docx]

ChCl:LAC

C -2.56902400 1.38754400 0.08994200

C -1.07663600 1.08890000 0.03024700

N -3.48320700 0.17048400 0.06546400

C -4.89710400 0.66618300 0.13139600

C -3.29520200 -0.62212000 -1.20804700

C -3.21818400 -0.72943100 1.25088000

O -0.45719900 2.36311300 0.06461000

H -2.79968100 1.93272700 1.00714000

H -2.85427900 2.00948000 -0.76061900

H -0.81549700 0.54333700 -0.87893400

H -0.76061000 0.46880300 0.87202400

H -5.57169200 -0.18825800 0.11533400

H -5.03461200 1.22983900 1.05314100

H -5.09038700 1.30731400 -0.72756100

H -4.07521000 -1.38174100 -1.24921700

H -2.31605800 -1.10845600 -1.18225200

H -3.38931600 0.05878300 -2.05337500

H -3.25776400 -0.12502400 2.15652800

H -3.99594400 -1.49210400 1.27429600

H -2.24375200 -1.20862200 1.12194100

H 0.49329400 2.18175200 0.00997000

Cl -0.44518800 -2.27370600 -0.13766100

C 4.96587500 -0.52503800 0.89828400

H 4.80619500 -1.60444500 0.89495300

H 4.61293800 -0.11353500 1.84735600

H 6.03474600 -0.32029100 0.81316000

C 4.23745500 0.13601400 -0.27417600

H 4.57481300 -0.32752800 -1.21100500

C 2.72591900 -0.08402600 -0.20568800

O 4.52424700 1.52265900 -0.31560300

O 2.39233000 -1.34812900 -0.22544200

H 1.39613700 -1.53735000 -0.19015500

O 1.95672500 0.86846300 -0.14989100

H 3.66991500 1.97791800 -0.29716400

BaCl:LAC

C -2.07145600 0.00482500 0.72019000

C -1.57923300 1.38180100 1.10996800

N -2.38564200 -0.18188400 -0.74731300

C -1.13037000 -0.03386000 -1.58821600

C -2.89303100 -1.59626800 -0.90678600

C -3.43930200 0.77911000 -1.21655100

O -1.39132600 1.40850800 2.43611700

O -1.39980000 2.32588100 0.38125300

H -1.31209400 -0.75448800 0.97599600

H -2.97602100 -0.22251200 1.28755900

H -1.41129700 -0.20770000 -2.62645700

H -0.73084100 0.96502500 -1.45633000

H -0.41202900 -0.77789900 -1.24959100

H -3.08792200 -1.76625600 -1.96474300

H -3.81407300 -1.70347500 -0.33401000

H -2.11905300 -2.27370600 -0.53600400

H -4.32233100 0.66738700 -0.58718700

H -3.04963600 1.79058800 -1.15051600

H -3.68955800 0.53340400 -2.24765700

H -0.97568400 2.25434400 2.66239400

Cl 0.07139600 -2.56605400 0.61294800

C 3.57075400 -0.83561600 0.98392800

H 4.60775900 -0.68470600 0.67878800

H 3.18904200 -1.75360100 0.53078100

H 3.53141700 -0.95300300 2.06836200

C 2.69670100 0.36218600 0.58244600

H 3.09414000 1.26588700 1.05915100

C 2.79119800 0.62152200 -0.92460900

O 1.36425300 0.23027900 1.00169400

O 4.04460300 1.01414500 -1.27385800

H 4.04433800 1.14889200 -2.23318700

O 1.90471000 0.51740000 -1.73447000

H 1.06852000 -0.70397800 0.88561900

EaCl:LAC

C 2.89018200 -1.40653600 -0.31104800

C 4.34940100 -1.01095600 -0.13803200

N 2.02181900 -0.71543500 0.68869400

H 1.01537500 -0.94712300 0.56932600

H 2.30714800 -0.91139700 1.64714600

H 2.02829100 0.35697900 0.52253200

H 2.51619600 -1.10774900 -1.29111200

H 2.74557600 -2.48341200 -0.20352600

H 4.95830800 -1.50664400 -0.89668900

H 4.73250100 -1.30913500 0.84267500

H 4.47568800 0.06796000 -0.24978200

Cl 1.60572700 2.11161400 -0.01826500

C -3.90329100 0.48073200 0.51599500

H -3.79827100 1.54694900 0.30979200

H -3.67554700 0.29401800 1.56835200

H -4.93590400 0.18030000 0.33095600

C -2.98083900 -0.34317200 -0.38619900

H -3.19752900 -0.09887200 -1.43515300

C -1.51190700 0.00408100 -0.16605600

O -3.19385300 -1.72709400 -0.17992500

O -1.23052900 1.25764500 -0.38577600

H -0.25350200 1.50414300 -0.24918500

O -0.71251300 -0.86895500 0.17762100

H -2.33454700 -2.11308400 0.04100600
